# Sex Differences in *Drosophila melanogaster* Heterochromatin Are Regulated by Non-Sex Specific Factors

**DOI:** 10.1371/journal.pone.0128114

**Published:** 2015-06-08

**Authors:** Manasi S. Apte, Victoria H. Meller

**Affiliations:** Department of Biological Sciences, Wayne State University, Detroit, Michigan, 48202, United States of America; Wellcome Trust Centre for Stem Cell Research, UNITED KINGDOM

## Abstract

The eukaryotic genome is assembled into distinct types of chromatin. Gene-rich euchromatin has active chromatin marks, while heterochromatin is gene-poor and enriched for silencing marks. In spite of this, genes native to heterochromatic regions are dependent on their normal environment for full expression. Expression of genes in autosomal heterochromatin is reduced in male flies mutated for the noncoding *roX* RNAs, but not in females. *roX* mutations also disrupt silencing of reporter genes in male, but not female, heterochromatin, revealing a sex difference in heterochromatin. We adopted a genetic approach to determine how this difference is regulated, and found no evidence that known X chromosome counting elements, or the sex determination pathway that these control, are involved. This suggested that the sex chromosome karyotype regulates autosomal heterochromatin by a different mechanism. To address this, candidate genes that regulate chromosome organization were examined. In XX flies mutation of *Topoisomerase II (Top2)*, a gene involved in chromatin organization and homolog pairing, made heterochromatic silencing dependent on *roX*, and thus male-like. Interestingly, Top2 also binds to a large block of pericentromeric satellite repeats (359 bp repeats) that are unique to the X chromosome. Deletion of X heterochromatin also makes autosomal heterochromatin in XX flies dependent on *roX* and enhances the effect of *Top2 mutations*, suggesting a combinatorial action. We postulate that Top2 and X heterochromatin in *Drosophila* comprise a novel karyotype-sensing pathway that determines the sensitivity of autosomal heterochromatin to loss of *roX* RNA.

## Introduction

Approximately 30% of the *Drosophila* genome is heterochromatic [1]. Many cytological and molecular features distinguish gene-poor heterochromatin from gene-rich euchromatin. Heterochromatin forms a compact, relatively inaccessible domain with ordered nucleosome arrays [2]. Heterochromatic loci tend to be near the nuclear periphery during interphase. Heterochromatin is characterized by repetitive DNA sequences, low levels of histone acetylation, hypomethylation at H3K4 and H3K79 and enrichment for Heterochromatin Protein 1 (HP1) [3]. Although relatively gene-poor, *Drosophila* heterochromatin harbors hundreds of protein coding genes (heterochromatic genes) [1, 4]. The native heterochromatic environment has been shown essential for full expression of some of these genes, and disruption of heterochromatin lowers their expression [5–7].

Euchromatic genes also rely on their native chromatin context, and stochastic silencing is observed when a euchromatic gene is placed in a heterochromatic environment, a phenomenon known as Position Effect Variegation (PEV). PEV represents variable spreading of inactivation over the euchromatic gene, producing irregular silencing [3]. PEV is extraordinarily sensitive to heterochromatin integrity. For example, mutation of a single copy of Su(Var)2–5, encoding HP1, elevates expression of variegating reporters inserted in heterochromatic regions. This effect, called suppression of PEV, enables identification of genes involved in heterochromatin formation and silencing.

*Drosophila* heterochromatin is typically not thought of as sexually dimorphic. However, recent studies suggest that heterochromatin in male and female flies differs. Reduction in HP1 results in preferential lethality and higher gene misregulation in males [8]. Mutation of the *Drosophila roX1* and *roX2* RNAs (*RNA on the X 1 and -2*) is a potent suppressor of PEV for autosomal insertions in male flies, but not in females [9]. A genome-wide reduction in the expression of autosomal heterochromatic genes is also observed in *roX1 roX2* males [9]. These findings suggest a general disruption of autosomal heterochromatin in *roX1 roX2* mutants that is limited to males. Sexually dimorphic heterochromatin could stem from differential sensitivity to reduced levels of factors necessary in both sexes, or by differences in the establishment or maintenance of heterochromatin in males and females. We refer to heterochromatin as masculine if *roX* RNA is necessary for normal PEV, and a feminine if *roX* is unnecessary. This designation does not require knowledge of the mechanism through which *roX* influences heterochromatin. Interestingly, the *roX* RNAs are also essential for X chromosome dosage compensation, another male-limited process [10]. *roX* RNAs assemble with the Male Specific Lethal (MSL) proteins to form a complex that is targeted to X-linked genes. Enzymatic activities within the MSL complex modify chromatin at X-linked genes, leading to increased transcription in male flies. Most of the MSL proteins are also required for full expression of autosomal heterochromatic genes in males [9]. The only member of the MSL complex that is unnecessary for heterochromatic genes is the Male Specific Lethal 2 (MSL2) protein. This is surprising as MSL2, a key regulator of X chromosome dosage compensation, is the sole member of the MSL complex with strictly male-limited expression. This raises intriguing questions about how the sexual dimorphism of heterochromatin is determined. We postulated that heterochromatic sex is under genetic control, and conducted experiments aimed at determining the signal that regulates this process.

Using a PEV reporter assay we demonstrated that feminization of heterochromatin is independent of female-limited components of the *Drosophila* sex determination pathway. Furthermore, neither MSL2 nor the Y chromosome directs heterochromatin masculinization. We then examined the numerator elements, components of the X chromosome counting mechanism, and saw no effect on heterochromatic sex. This suggests that a novel signal, perhaps direct sensing of karyotype, could be involved. As flies pair homologous chromosomes, the sex chromosome karyotype could be detected by the presence of unpaired chromatin in XY or XO flies. Screening of viable mutations that influence chromosome organization and homologue pairing revealed that *Topoisomerase II* (*Top2*) contributes to the feminization of autosomal heterochromatin in XX flies. Top2 promotes homologue pairing, consistent with pairing-dependent detection of sex chromosome karyotype. However, Top2 also binds satellite repeats that make up over 10 Mb of pericentric X heterochromatin [11]. Interestingly, loss of X-heterochromatin partially masculinizes autosomal heterochromatin in XX flies also. We propose that *Top2* and pericentromeric X heterochromatin together control the sexual differentiation of heterochromatin in *Drosophila melanogaster*. The ubiquity of Top2 and repetitive sequences suggests a general mechanism for direct detection of karyotype.

## Results

Two metrics of autosomal heterochromatic integrity are disrupted in *roX1 roX2* (*roX*) males, but not females. First, expression of heterochromatic genes on the autosomes decreases in male larvae carrying the severely affected *roX1* ^*SMC17A*^ *roX2Δ* chromosome [9]. Second, adult male escapers with the partial loss of function *roX1* ^*ex33*^ *roX2Δ* chromosome display a dramatic suppression of PEV at autosomal insertions. However, no suppression of PEV or reduction in heterochromatic gene expression is detected in females with these *roX* mutations. These observations were surprising because the *roX* RNAs were not thought to play a role outside of X chromosome dosage compensation. In addition, autosomal heterochromatin is not overtly sexually dimorphic. Variegating insertions typically behave similarly in males and females, and the autosomal heterochromatic genes that are misregulated in *roX* males rarely display sex-biased expression [9]. The underlying cause of the differences in male and female heterochromatin is completely unknown. In this study, we used a genetic approach to examine this question.

**Fig 1:**
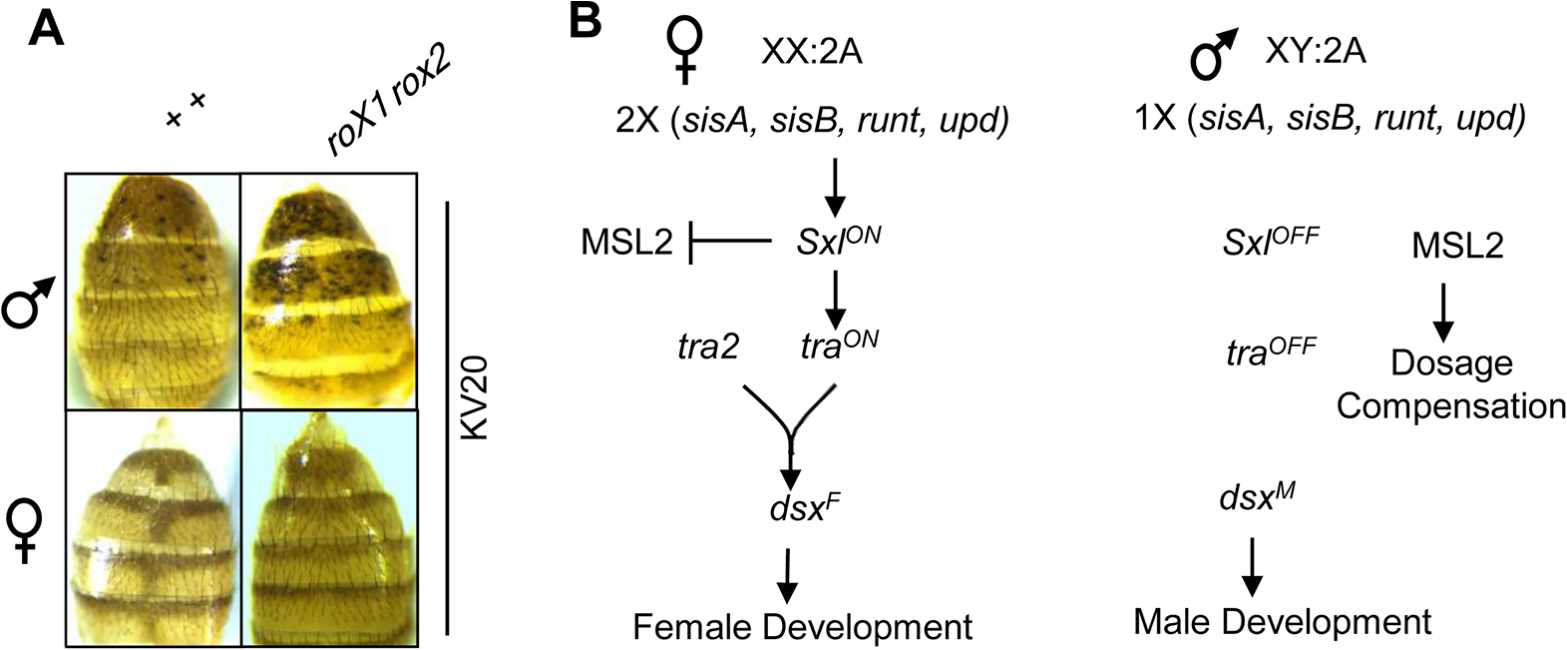
Heterochromatin masculinization is revealed by position effect variegation (PEV). (A) PEV of a *y* ^*+*^ marker in the KV20 insertion produces black abdominal spots. Suppression of PEV in *yw roX1* ^*ex33*^ *roX2Δ* /Y; KV20/+ males increases pigmentation (top). Females (bottom) typically produce less than one spot per female, and no suppression of PEV is detected in *yw roX1* ^*ex33*^ *roX2Δ*; KV20/+ females (right). (B) Somatic sex determination in flies is controlled by the number of X chromosomes. Two copies of X-linked numerator elements (*sisA*, *sisB*, *runt* and *upd*) turn on *Sexlethal (Sxl)* expression in XX embryos. *Sxl* blocks dosage compensation by preventing translation of MSL2 in XX embryos. *Sxl* ensures productive splicing of *transformer* (*tra*) mRNA. *tra* and *transformer2* (*tra2*) induce the female-specific isoform of *doublesex* (*dsx* ^*F*^). Only *dsx* ^*M*^ is produced in males. The Dsx transcription factors coordinate visible somatic differentiation. Additional *tra* and *tra2* targets (not shown) regulate differentiation of the nervous system.

Suppression of PEV increases black abdominal pigmentation from variegating *y* ^*+*^ reporters (Fig 1A, S1A Fig) and red eye pigmentation from variegating *w* ^*+mW*.*hs*^ reporters (S1B Fig). The 3^rd^ chromosome insertion KV24 displays *y* ^*+*^ PEV in both sexes and the 2^nd^ chromosomal insertion KV20 displays PEV in males, but typically produces less than 1 spot on each female abdomen. Suppression of PEV in *roX1* ^*ex33*^ *roX2Δ* males was observed for all the autosomal insertions tested, but no effect was observed in *roX1* ^*ex33*^ *roX2Δ* females, revealing an effect that is not unique to a specific insertion site or reporter (Fig 1A, S1 Fig and [9]).

To understand how this difference in fly heterochromatin arises, we conducted a screen for the genetic determinants of heterochromatin sexual dimorphism. This screen encompassed the sex determination pathway as well as elements of the sex chromosome karyotype. Matched genotypes differing only at the *roX* genes were generated to determine if heterochromatin is masculine (*roX1 roX2* mutation suppresses PEV) or feminine (no or minor suppression of PEV in *roX1 roX2* mutants) in each genetic background. *Drosophila* sex determination is triggered by the X chromosome dose (X:A, Fig 1B). The Y chromosome is believed to have no role in *Drosophila* sex determination. The two X chromosomes in female embryos initiate early expression of *Sexlethal* (*Sxl*) [12]. Sxl induces productive *transformer* (*tra*) splicing [13]. Tra and Transformer 2 (Tra2) direct splicing of the female isoform of the *doublesex* transcription factor (*dsx* ^*F*^). Conversely, in XY embryos *Sxl* is not expressed [14, 15]. Sxl represses MSL2 translation [16–18]. As MSL2 is a key protein in X chromosome dosage compensation, this limits dosage compensation to males. The absence of Sxl in males also prevents *tra* expression, resulting in the production of default male isoform of *dsx* (*dsx* ^*M*^). We hypothesized that genes in the sex determination pathway, or the Y chromosome, might control the observed sexual dimorphism of heterochromatin.

**Fig 2:**
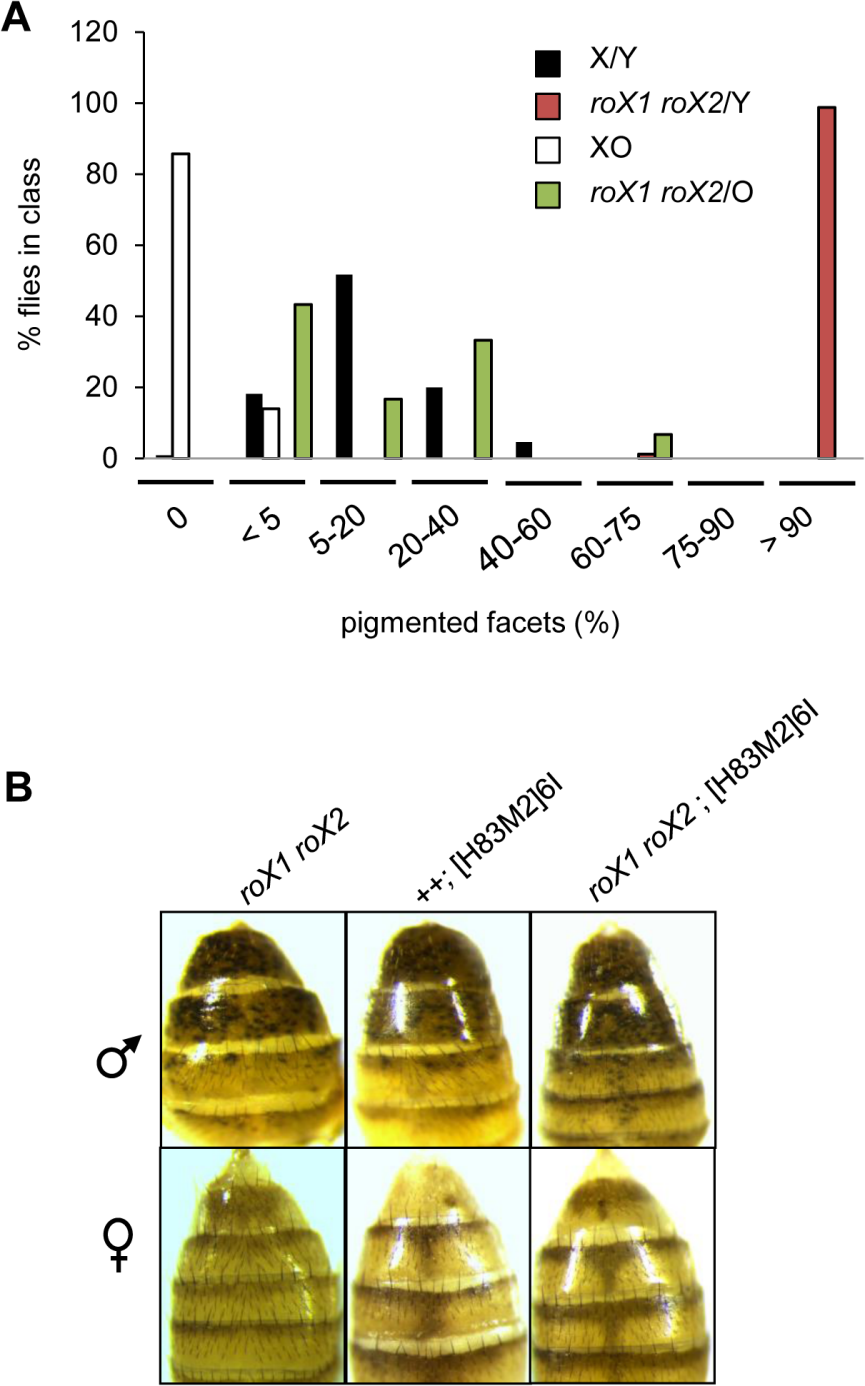
Neither the Y chromosome nor MSL2 direct heterochromatin masculinization. (A) Eye pigmentation was examined in flies with a variegating marker (*w* ^*+mW*.*hs*^) in the 118E-10 insertion. In XY males (black and red bars) loss of *roX* (red) dramatically suppresses PEV. XO males display stronger silencing (white and green bars) but loss of *roX* (green) still suppresses PEV. Full genotypes and number of individuals scored are: black, *yw*/Y; 118E-10/+, 110, white, *yw*/O; 118E-10/+, 21, red, *yw roX1* ^*ex33*^ *roX2Δ* /Y; 118E-10/+, 83 and green, *yw roX1* ^*ex33*^ *roX2Δ* /O; 118E-10/+, 30. (B) MSL2 does not masculinize XX heterochromatin. Ectopic MSL2 expression from the [H83M2]6I transgene does not lead to suppression of PEV in *yw roX1* ^*ex33*^ *roX2Δ* females. PEV of *y* ^*+*^ KV20 is suppressed in *yw roX1* ^*ex33*^ *roX2Δ* males, and remains unchanged by increased MSL2 expression. At least 50 flies were scored per genotype. Representative male (top) and female (bottom) adults are presented.

We first considered the possibility that a male-limited factor masculinizes heterochromatin. The Y chromosome is thought to act as a sink for heterochromatin proteins, and thus has epigenetic effects throughout the genome [19, 20]. We generated males with a variegating *w* ^*+mW*.*hs*^ marker (insertion 118E-10) that were wild type for the *roX* genes or carried the partial loss of function *roX1* ^*ex33*^ mutation and a deletion of *roX2*, a combination that allows over 20% escaper males. Eyes of control males (*yw*/Y; 118E-10/+) have an average of 20% pigmented facets (black bars, Fig 2A), but *yw roX* ^*ex33*^ *roX2*/Y; 118E-10/+ males display over 90% pigmentation, a dramatic suppression of PEV (red bars, Fig 2A). The absence of a Y chromosome in XO males frees heterochromatin proteins to reinforce silencing and enhance PEV at other loci [20]. As expected, PEV was enhanced in XO males with wild type *roX* genes, almost 90% of which have no eye pigmentation (*yw*/O; 118E-10/+; white bars in Fig 2A). We then asked whether PEV in XO males was suppressed by *roX* mutations, and found that all *yw roX* ^*ex33*^ *roX2/O*; 118E-10/+ males display at least some eye pigmentation (green bars in Fig 2A). Since the loss of *roX* suppresses PEV in otherwise identical XO males (compare white and green bars in Fig 2A), we conclude that the presence of the Y chromosome is not responsible for masculine heterochromatin in males.

The protein Male Specific Lethal-2 (MSL2) binds the *roX* RNAs and is the only male-limited member of the dosage compensation complex [21–23]. To determine if MSL2 plays a role in heterochromatin masculinization, we expressed MSL2 from the [H83M2]6I transgene in XX females with a variegating *y* ^+^ reporter (insertion KV20), and compared females that were either wild type or mutated for the *roX* genes [23–25]. This, and following studies utilize *roX2Δ* a simple deletion that facilitates stock construction [26]. PEV in females expressing MSL2 is not influenced by *roX* mutations (Fig 2B, bottom). In contrast, *roX* mutations suppress PEV in males of matched genetic background (Fig 2B, top). This is consistent with a study finding that MSL2 is not required for full expression of autosomal heterochromatic genes in males [9]. As MSL2 appears to have no role in either measure of sexually dimorphic heterochromatin, we conclude that it does not masculinize heterochromatin.

Loss of *roX* RNAs in males leads to relocalization of MSL proteins to the chromocenter, a structure composed of pericentromeric heterochromatin from all chromosomes. Identical MSL mislocalization is also observed in *roX1 roX2* females that ectopically express MSL2 [9]. In spite of the abnormal recruitment of MSL proteins to the chromocenter, no disruption of heterochromatic gene expression or PEV can be detected in *roX1 roX2* females that ectopically express MSL2 (Fig 2B and [9]). We conclude that mislocalization of MSL proteins does not produce the disruptions in heterochromatin function that are observed in *roX1 roX2* mutants.

**Fig 3:**
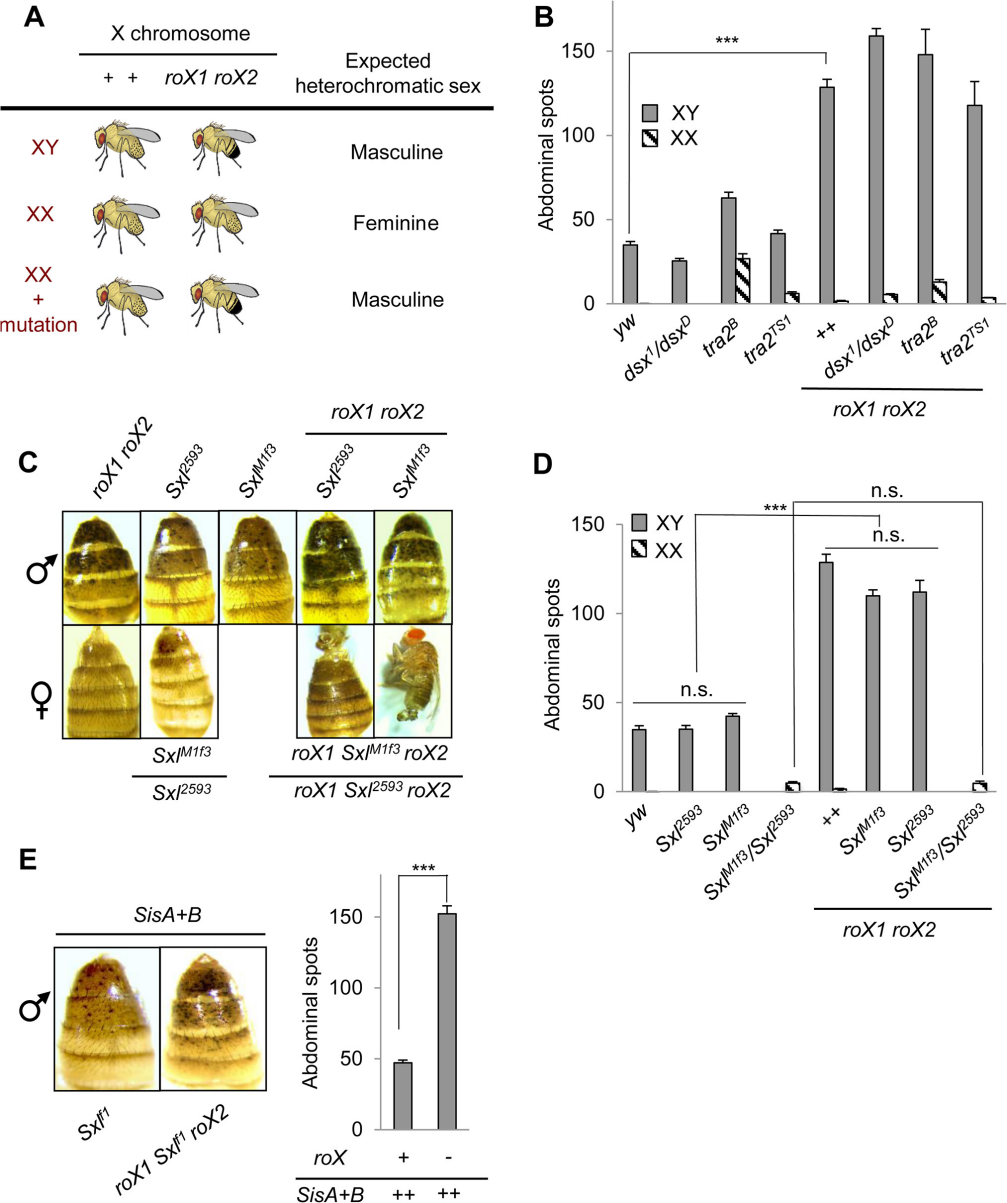
The somatic sex determination pathway and numerator elements do not control heterochromatin feminization. (A) Scheme for identification of genetic regulators of heterochromatic sex. Heterochromatin is masculine if loss of *roX* suppresses PEV of an autosomal reporter. If a gene in the sex determination cascade normally feminizes XX heterochromatin, mutation of that gene will masculinize XX heterochromatin, leading to suppression of PEV in *roX* mutants. (B) *tra2* and *dsx* do not feminize heterochromatin. *yw roX1* ^*ex33*^ *roX2Δ* / *B* ^*s*^ *Y*; KV20/+ males with *tra2* ^*B*^, *tra2* ^*ts1*^ or *dsx* ^*1*^ */dsx* ^*D*^ mutations display suppression of PEV, detected by increased abdominal pigmentation (gray bars at right). XX pseudomales and intersexes display a modest increase in spots, consistent with masculinization of pigmentation patterns (hatched bars). However, no suppression of PEV is observed in *roX* pseudomales. Full genotypes (left to right) are: *yw;* KV20/+, *yw;* KV20/+; *dsx* ^*1*^ */dsx* ^*D*^, *yw; tra2* ^*B*^ KV20/ *tra2* ^*B*^, *yw; tra2* ^*TS1*^ KV20/ *tra2* ^*TS1*^, *yw roX1* ^*ex33*^ *roX2Δ*; KV20/*+*, *yw roX1* ^*ex33*^ *roX2Δ*; KV20/*+; dsx* ^*1*^ */dsx* ^*D*^, *yw roX1* ^*ex33*^ *roX2Δ*; *tra2* ^*B*^ KV20/ *tra2* ^*B*^, *yw roX1* ^*ex33*^ *roX2Δ*; *tra2* ^*TS1*^ KV20/ *tra2* ^*TS1*^). Twenty-50 individuals of each genotype were scored. (C) *Sxl* mutations do not masculinize XX heterochromatin. Representative XY (top) and XX (bottom) flies are shown. XY flies with *Sxl* mutations suppress PEV upon loss of *roX* function (right two panels). XX *Sxl* ^*M1*,*f3*^ */Sxl* ^*2593*^ pseudomales display partial masculinization of genitalia and pigmentation, but no suppression of PEV is observed upon *roX* mutation. (D) Abdominal pigmentation in *Sxl* adults. Full genotypes of XY flies (gray bars): *yw*/Y; KV20/+, 75 flies, *yw Sxl* ^*2593*^ /Y; KV20/+, 75 flies, *yw Sxl* ^*M1*,*f3*^/Y; KV20/+, 64 flies, *yw roX1* ^*ex33*^ *Sxl* ^*M1*,*f3*^ *roX2Δ*/Y; KV20/+, 17 flies, *yw roX1* ^*ex33*^ *Sxl* ^*2593*^ *roX2Δ* Y KV20/+, 37 flies. Full genotypes of XX flies (hatched bars): *yw Sxl* ^*M1*,*f3*^ */ yw Sxl* ^*2593*^ KV20/+, 21 flies, *yw roX1* ^*ex33*^ *Sxl* ^*M1*,*f3*^ *roX2Δ/ yw roX1* ^*ex33*^ *Sxl* ^*2593*^ *roX2Δ* KV20/+, 10 flies. (*p*-value ***<0.00001, n.s = non-significant). (E) Numerator elements do not feminize XY heterochromatin. Overexpression of *SisA* and *SisB* is indicated by ++. Full genotypes: *ywSxl* ^*f1*^/Y; 2X*P(w* ^+mC^,*sisA* ^+^)+ 2X*P(w* ^+mC^,*sc* ^sisB+^)/KV20 and *yw roX1* ^*ex33*^ *Sxl* ^*f1*^ *roX2Δ*/Y; 2X*P(w* ^+mC^,*sisA* ^+^) +2X*P(w* ^+mC^,*sc* ^sisB+^)/KV20. Data was derived from over 20 individuals per genotype. *** indicates *p*-value <0.00001.

We then addressed the possibility that female-limited proteins in the somatic sex determination pathway feminize autosomal heterochromatin. If this is the case, mutations in this pathway will masculinize heterochromatin in XX flies (Fig 3A). We tested *Sexlethal (Sxl)*, *tranformer2 (tra2)* and *doublesex* (*dsx*), representing different levels in the sex determination hierarchy (Fig 1B, left). As these genes direct female somatic differentiation, mutations produce XX intersexes or pseudomales with male-like body pigmentation and altered genital morphology. *dsx* ^*1*^ is amorphic and *dsx* ^*D*^ produces the male splice form. XX; *dsx* ^*1*^ */dsx* ^*D*^ flies are fully masculinized. We generated X/Y; *dsx* ^*1*^ */dsx* ^*D*^ and XX; *dsx* ^*1*^ */dsx* ^*D*^ flies with KV20 and the *yw roX1* ^*ex33*^ *roX2Δ* chromosome. Masculinized XX; *dsx* ^*1*^ */dsx* ^*D*^ flies were distinguished from XY flies by the absence of a marked Y chromosome (*B* ^*s*^ *Y)*. Masculinization increased abdominal pigmentation, allowing detection of more *y* ^*+*^ spots in XX flies. Because of this, comparisons must be between flies with the same *dsx* status. Although *yw roX1* ^*ex33*^ *roX2Δ*/ *B* ^*s*^ *Y*; KV20/+; *dsx* ^*1*^ */dsx* ^*D*^ males displayed strong suppression of PEV in comparison to males with wild type *roX*, no suppression of PEV was observed in XX; *dsx* ^*1*^ */dsx* ^*D*^ pseudomales upon loss of *roX* (compare *yw roX1* ^*ex33*^ *roX2Δ*; KV20/+; *dsx* ^*1*^ */dsx* ^*D*^ and *yw*; KV20/+; *dsx* ^*1*^ */dsx* ^*D*^, Fig 3B).

We next tested the *tra2* ^*ts1*^ and *tra2* ^*B*^ mutations. *tra2* ^*ts1*^ is a temperature sensitive hypomorph and *tra2* ^*B*^ is a null allele. Loss of *tra2* has no visible effect on XY flies but masculinizes XX flies. We generated XX and XY *tra2* mutants carrying KV20 and *yw roX1* ^*ex33*^ *roX2Δ*. Loss of *roX* suppressed PEV in *tra2/ tra2* males (Fig 3B). In contrast, XX; *tra2/ tra2* pseudomales mutated for *roX* displayed no suppression of PEV (Fig 3B).

Although *dsx* and *tra2* do not regulate heterochromatin sexual differentiation, it remained possible that *Sxl*, the master regulator of sexual determination, did so through a different pathway. Since null *Sxl* mutations are embryonic lethal in XX zygotes, we tested a heteroallelic combination, *Sxl* ^*M1*,*f3*^ */Sxl* ^*2593*^, that produces masculinized XX adult escapers. The *roX* genes and *Sxl* are X-linked, necessitating generation of two *roX1* ^*ex33*^ *Sxl roX2Δ* chromosomes. Control masculinized XX adults (*yw Sxl* ^*M1*,*f3*^ */ yw Sxl* ^*2593*^; KV20/+) emerged late and displayed developmental defects and partial sexual transformation (Fig 3C, bottom). Similar to XX flies masculinized by *tra2* and *dsx*, a few abdominal spots were visible. However, mutation of *roX* had no effect on PEV in XX flies that were masculinized by *Sxl* mutations (Fig 3D, hatched bars). In contrast, XY males mutated for *roX* and *Sxl* displayed strong suppression of PEV (Fig 3C and 3D). This supports the idea that sexual differentiation of heterochromatin is independent of the somatic sex determination pathway. One caveat is that this test requires adult escapers, preventing use of null *Sxl* alleles. It remains possible that a novel *Sxl* function is retained in the heteroallelic combination tested. Sxl regulates *roX1* expression by repression of MSL2 [27, 28]. One possibility is that Sxl regulates heterochromatic sexual differentiation by modulating *roX1* levels in early embryos. For example, high *roX1* RNA concentrations could establish male heterochromatin. Repression of MSL2 by Sxl in females reduces *roX1* levels. Arguing against this idea is the observation that ectopic expression of MSL2 fails to masculinize heterochromatin. Furthermore, *roX1* is abundant in early embryos of both sexes, and pseudomales generated using a similar heteroallelic *Sxl* combination have elevated *roX1* levels[29][30]. However, none of these manipulations activate dosage compensation or *roX1* expression to the level observed in normal males. Nevertheless, the stability of heterochromatic sex in genetic backgrounds mutated for *tra* and *dsx* suggests genetic regulation at the level of *Sxl* or above.

A mechanism that detects sex chromosome karyotype could bypass the sex determination cascade altogether. One way this could occur is if the X chromosome counting mechanism that turns on *Sxl* in XX embryos also controls a second pathway that leads to heterochromatin feminization. Proteins from the X-linked *sisterless A and B* (*sisA* and *sisB)*, *unpaired (upd*) and *runt (runt*) genes, collectively known as numerator elements, promote early *Sxl* expression in XX embryos [31–34]. Elevated *sisA* and *sisB* expression is benign in XX flies but turns on *Sxl* expression in XY flies, a lethal situation that can be overcome by mutating *Sxl* [35, 36]. We examined heterochromatin sexual differentiation in XY flies with multiple *sisA* and *sisB* transgenes and the *Sxl* ^*f1*^ mutation. We found normal PEV in control males that have wild type *roX* and overexpress *sisA* and *sisB*, but strong suppression of PEV when *roX* mutations are introduced into this genotype, revealing stable heterochromatin masculinization (Fig 3E). We conclude that *sisA* and *sisB*, key components of the X chromosome counting mechanism, do not feminize heterochromatin.

Another possible mechanism for detection of karyotype involves chromosome pairing. Interphase chromosomes of *Drosophila* are paired throughout development [37–39]. All homologs pair in females, but the structurally dissimilar X and Y chromosomes of males remain unpaired. In theory, unpaired chromatin in XY and XO cells could signal the male karyotype.

To investigate this possibility, we examined several genes that regulate homolog pairing in *Drosophila* [39, 40]. Three pairing promoters, *Topoisomerase II* (*Top2*), *Dynein Heavy chain-64c* (*Dhc64c*) and *Microcephalin-1* (*MCPH1*), and three anti-pairers, c*ondensin II* subunits *Cap-H2* and *Cap-D3*, and *Female sterile (1) homeotic* (*fs(1)h*) were examined. Some of these are essential, requiring the use of partial loss of function mutations, or heteroallelic combinations that produce adult escapers. HP1, an anti-pairing gene, was not selected for the screen, as mutation of HP1 is a potent suppressor of PEV regardless of sex. If fully paired chromosomes signal the XX karyotype, and this in turn regulates heterochromatic sex, mutation of anti-pairers will increase pairing, leading to feminization of autosomal heterochromatin in XY animals. We generated XX and XY flies with KV20 and viable mutations in individual anti-pairers. Each was constructed with wild type or mutated *roX* genes. Abdominal spots were minimal, but unchanged, in *roX* mutant females. Males with *Cap-H2* ^*Z0019*^, *Cap-D3* ^*c07081*^ or *fs(1)h* ^*1*^ mutations continued to suppress PEV when mutated for *roX* (S2 Fig, compare gray and black bars). We conclude that mutation of these anti-pairing factors does not lead to feminization of heterochromatin in males.

We then tested mutations in pairing promoters. These mutations reduce pairing, a condition that could mimic the unpaired chromatin of males. If unpaired chromatin signals the XY karyotype, reduced pairing in XX flies could inappropriately masculinize heterochromatin. We first generated individual XX and XY flies with loss of function mutations in *Dhc64c* or *MCPH1*, KV20, and wild type or mutated for the *roX* genes. XY flies mutated for *Dhc64c* or *MCPH1* continued to show suppression of PEV when mutated for *roX* (*yw roX1* ^*ex33*^ *roX2ΔY*; *MCPH1* ^*0978*^ KV20 / *MCPH1* ^*0978*^ and *yw roX1* ^*ex33*^ *roX2ΔY*; KV20 /*+; dhc64c* ^*6-10*^ */ dhc64c* ^*8-1*^) (S2 Fig, gray bars). However, no masculinization of heterochromatin was apparent in females mutated for *Dhc64c* or *MCPH1* (S2 Fig, hatched bars).

**Fig 4:**
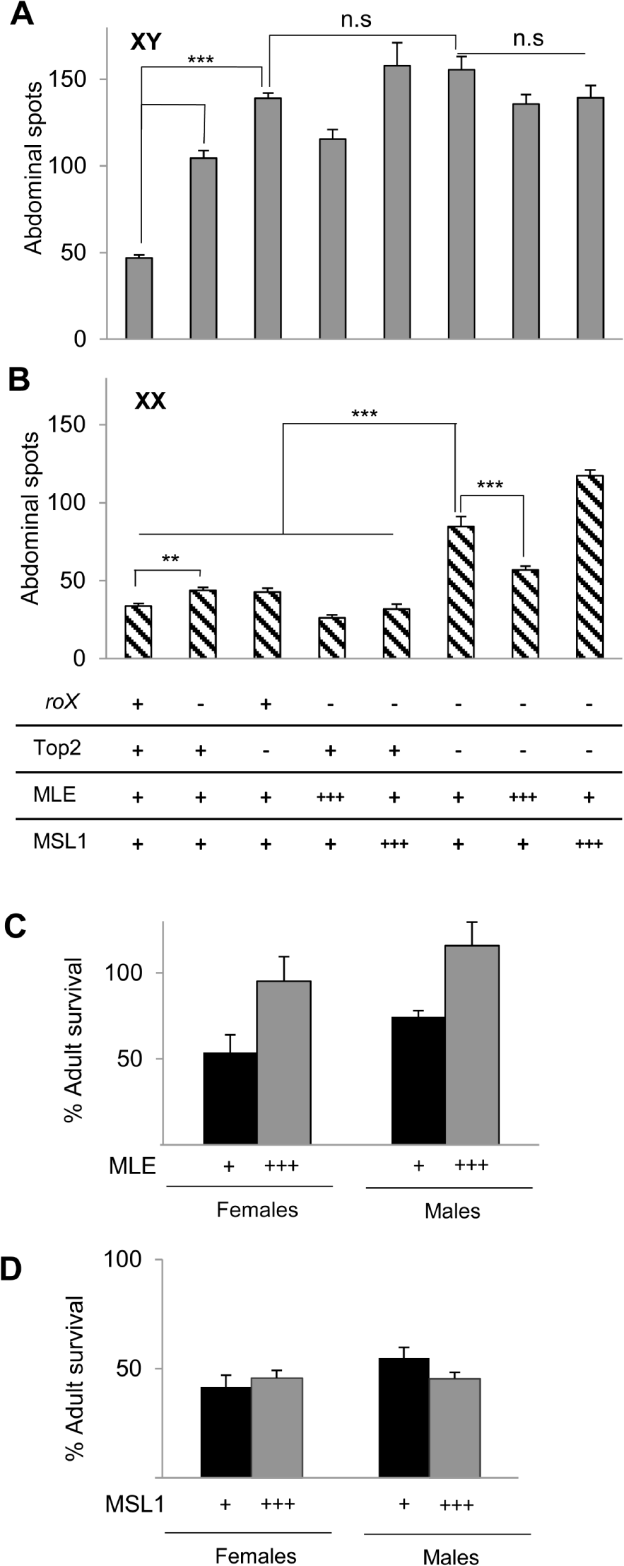
Mutation of *Topoisomerase II (Top2)* masculinizes XX heterochromatin. (A) PEV is suppressed in males mutated for *roX* or *Top2*. Ectopic MLE or MSL1 expression does not restore PEV in *roX* or *Top2* mutants. Twenty-50 flies of each genotype were scored. *p*-values: ** <0.0001; *** <0.00001; n.s non-significant. (B) Suppression of PEV in *Top2* females mutated for *roX*. Pigmentation displays little or no increase in XX flies mutated for *roX* or Top2 alone (left three bars). Simultaneous mutation of *roX* and Top2 leads to suppression of PEV. Over expression of MLE, but not MSL1, partially restores PEV in *roX* and *Top2* females (right two bars). (for A, B) Wild type (+) and mutant (-) for indicated genes; (+++) overexpressing transgenes. Full genotypes (left to right) *yw*; KV24/+, *yw roX1* ^*ex33*^ *roX2Δ*; KV24/+, *yw*; *Top2* ^*17-1*^ */Top2* ^*17-3*^; KV24/+, *yw roX1* ^*ex33*^ *roX2Δ*; [H83MLE]/+; KV24/+, *yw roX1* ^*ex33*^ *roX2Δ*; +/+; KV24/[H83M1]Z1, *yw roX1* ^*ex33*^ *roX2Δ*; *Top2* ^*17-1*^ */Top2* ^*17-3*^; KV24/+, *yw roX1* ^*ex33*^ *roX2Δ*; *Top2* ^*17-1*^ */Top2* ^*17-3*^ [H83MLE]; KV24/+, *yw roX1* ^*ex33*^ *roX2Δ*; *Top2* ^*17-1*^ */Top2* ^*17-3*^; KV24/[H83M1]Z1. (C) Overexpression of MLE rescues *Top2* lethality in both sexes. *yw*; *Top2* ^*17-1*^/*CyO y* ^*+*^ females were mated to *yw*; *Top2* ^*17-3*^/*CyO y* ^*+*^ or *yw*; *Top2* ^*17-3*^[H83 MLE] /*CyO y* ^*+*^ males. Survival of *yw*;*Top2* ^*17-1*^/ *Top2* ^*17-3*^ (black) and *yw*; *Top2* ^*17-1*^/ *Top2* ^*17-3*^[H83 MLE] (gray) was calculated by setting recovery of flies with *CyOy* ^*+*^ to 100%. Data was compiled from at least 3 replicate matings. (D) Overexpression of MSL1 does not rescue *Top2* ^*17-1*^/ *Top2* ^*17-3*^ survival. *yw; Top2* ^*17-1*^/ In(2LR)*GlaBc* females were mated to *yw*/Y; *Top2* ^*17-3*^/In(2LR)*GlaBc*; [H83M1]Z1/+ males. Survival of *yw*;*Top2* ^*17-1*^/ *Top2* ^*17-3*^ (black) and *yw*;*Top2* ^*17-1*^/*Top2* ^*17-3*^; [H83M1]Z1/+ (gray) was calculated by setting recovery of flies with In(2LR)*GlaBc* to 100%. Survival is derived from 5 replicate matings.

We then tested *Top2*, a pairing promoter with critical roles in nuclear organization, cell division and DNA repair. Since loss of *Top2* is lethal, the complementing heteroallelic *Top2* ^*17-1*^ */Top2* ^*17-3*^ combination was used [41]. Each mutation is individually lethal, but *Top2* ^*17-1*^ */Top2* ^*17-3*^ flies display >50% viability. *Top2* ^*17-1*^ (S791F) in the WHD domain reduces protein accumulation, but *Top2* ^*17-3*^ (L471Q) in the TOPRIM domain produces stable, full-length protein (S3A Fig). We generated *Top2* ^*17-1*^ */Top2* ^*17-3*^ XX and XY flies with variegating *y* ^*+*^ (KV24 insertion) that were in addition either wild type or mutated for the *roX* genes. The switch to the 3^rd^ chromosome KV24 was necessitated by our inability to recover a recombinant second chromosome with KV20 and *Top2*. We observed that *Top2* ^*17-1*^ */Top2* ^*17-3*^ itself suppressed PEV in males, but not in females, thus identifying an additional difference in the heterochromatin of males and females (Fig 4A and 4B). Surprisingly, *Top2* ^*17-1*^ */Top2* ^*17-3*^ females displayed highly significant suppression of PEV upon loss of *roX*, suggesting masculinization of XX heterochromatin by *Top2* mutation (Fig 4B). However, mutation of *Top2* does not otherwise sexually transform XX flies, which display female morphology.

Top2 was the sole pairing promoter that altered the sexual differentiation of heterochromatin, raising questions about the precise molecular function that is disrupted by the mutations used. *Top2* ^*17-1*^ */Top2* ^*17-3*^ males are fertile, but embryos deposited by *Top2* ^*17-1*^ */Top2* ^*17-3*^ females fail to hatch (S3B Fig). No evidence of DNA replication could be detected in these embryos by DNA staining (not shown), consistent with meiotic or mitotic failure [42]. We conclude that meiosis, fertilization or embryonic development of *Top2* ^*17-1*^ */Top2* ^*17-3*^ mutants requires maternal provision of wild type Top2.

We then examined polytene preparations from wild type and *Top2* ^*17-1*^ */Top2* ^*17-3*^ larvae to determine if there was a visible effect on chromosome organization. Similar heteroallelic *Top2* mutants have been shown to disrupt the male X-chromosome [41]. We scored chromosome morphology as abnormal if banding was diffuse and puffy if the chromosome was bloated along its entire length. Chromosomes from *Top2* mutants are more susceptible to breaking, suggesting fragility. Seventy percent of male nuclei from *Top2* mutants had abnormal or puffy X chromosomes (S3C Fig, black arrows), but only 14% of X chromosomes from wild-type males were scored as abnormal. *Top2* mutant females and wild type females display similar levels of X chromosome abnormality (10–15%). Fifty percent of nuclei from *Top2* mutants had partially unpaired homologs, in contrast to 15% from wild type larvae (S3C Fig, white arrows, S1 Table). The size, position and extent of unpairing varied between nuclei, and unpaired regions were equally prevalent in males and females. As most of the genome remains paired, this defect appears relatively minor. In summary, examination of chromosomes suggests selective disruption of male X-chromosome polytenization in *Top2* mutant larvae and homolog pairing that remains largely intact.

We then examined homolog pairing using a genetic assay. Pairing enables enhancers from one mutant allele to drive the promoter of a different allele, thus restoring expression (transvection). Transvection at *yellow (y)* is detected by increased pigmentation. While *y* ^*82f29*^ is a deletion of upstream enhancer elements, *y* ^*1#8*^ retains enhancers but lacks a promoter. Transvection in *y* ^*82f29*^ */y* ^*1#8*^ flies restores body, wing and bristle color (S3D Fig). *y* ^*3c3*^ lacks a bristle enhancer and the *y* promoter, but retains a wing enhancer. Transvection in *y* ^*82f29*^ */y* ^*3c3*^ flies restores wing pigmentation (S3 Fig). Flies homozygous for any one of these alleles have light bodies, wings and bristles. Heteroallelic *y* ^*82f29*^ */y* ^*1#8*^ and *y* ^*82f29*^ */y* ^*3c3*^ flies in wild type and *Top2* ^*17-1*^ */Top2* ^*17-3*^ mutant backgrounds displayed equivalent transvection (S3D and S3E Fig). We conclude that *Top2* ^*17-1*^ */Top2* ^*17-3*^ mutants retain sufficient homolog pairing to support transvection at *y*. Although no defect in *y* pairing was observed by this test, it is formally possible that the *Top2* mutants we tested are defective for pairing at other loci.

The *y* ^*2*^ allele is produced by a *Gypsy* insulator that prevents wing and body enhancers from contacting the promoter. Top2 is necessary for *Gypsy* insulation, and loss of Top2 restores pigmentation in the wing and body of *y* ^*2*^ flies [43]. We examined insulator function by comparing pigmentation in *y* ^*2*^ males that are wild type and *Top2* ^*17-1*^ */Top2* ^*17-3*^. No increase in body or wing color could be detected in *y* ^*2*^/Y; *Top2* ^*17-1*^ */Top2* ^*17-3*^ flies (S3F Fig). We conclude that the *Top2* ^*17-1*^ */Top2* ^*17-3*^ flies retain *Gypsy* insulator function, consistent with tests of other viable heteroallelic *Top2* combinations [44].

*Top2* was recently reported to participate in dosage compensation [45]. In support of this idea, a physical interaction between Top2 and Maleless (MLE), an RNA helicase that is a member of the dosage compensation complex, was detected. Based on this, and the disruption of X chromosome morphology in male *Top2* ^*17-1*^ */Top2* ^*17-3*^ mutants, we asked whether *Top2* ^*17-1*^ */Top2* ^*17-3*^ affects males more strongly than females. Interestingly, *Top2* ^*17-1*^ */Top2* ^*17-3*^ flies do not display male-preferential lethality, suggesting that these mutations do not affect the dosage compensation function of Top2 (Fig 4C, black bars). The association between Top2 and MLE prompted us to ask whether overexpression of MLE from a transgene [H83 MLE] could influence the survival of *Top2* ^*17-1*^ */Top2* ^*17-3*^ flies. MLE overexpression dramatically rescued *Top2* ^*17-1*^ */Top2* ^*17-3*^ mutants of both sexes (Fig 4C, gray). However, no rescue of Top2 mutants was achieved by overexpression of another member of the dosage compensation complex, *male-specific lethal 1* (*msl1*) (Fig 4D). Our data supports the idea of an interaction between Top2 and MLE, but the lack of sex-specificity of rescue argues against a role that is limited to dosage compensation.

The increased survival of *Top2* ^*17-1*^ */Top2* ^*17-3*^ mutants upon MLE overexpression prompted us to ask if MLE could restore heterochromatin function in Top2 mutants. To address this we generated *Top2* ^*17-1*^ */Top2* ^*17-3*^ mutants that overexpress MLE, carry the KV24 reporter and are either wild type or mutant for the *roX* genes. Increased MLE expression failed to restore PEV in males mutated for *roX* and Top2 (Fig 4A). In contrast, expression of MLE in *roX* and *Top2* mutant females achieved significant restoration of PEV (Fig 4B). However, overexpression of MSL1 failed to restore PEV in *roX* and Top2 mutant females (Fig 4B). Taken together, these findings support the idea that a *Top2*—MLE interaction is necessary for a process other than compensation, but the basis for the sex-specific effect of MLE on restoration of female PEV is speculative at present. However, MLE is part of the MSL complex, making it plausible that recruitment of MLE to the male X chromosome reduces its availability for interaction with Top2 on autosomal heterochromatin, producing the observed differences in response to overexpression.

**Fig 5:**
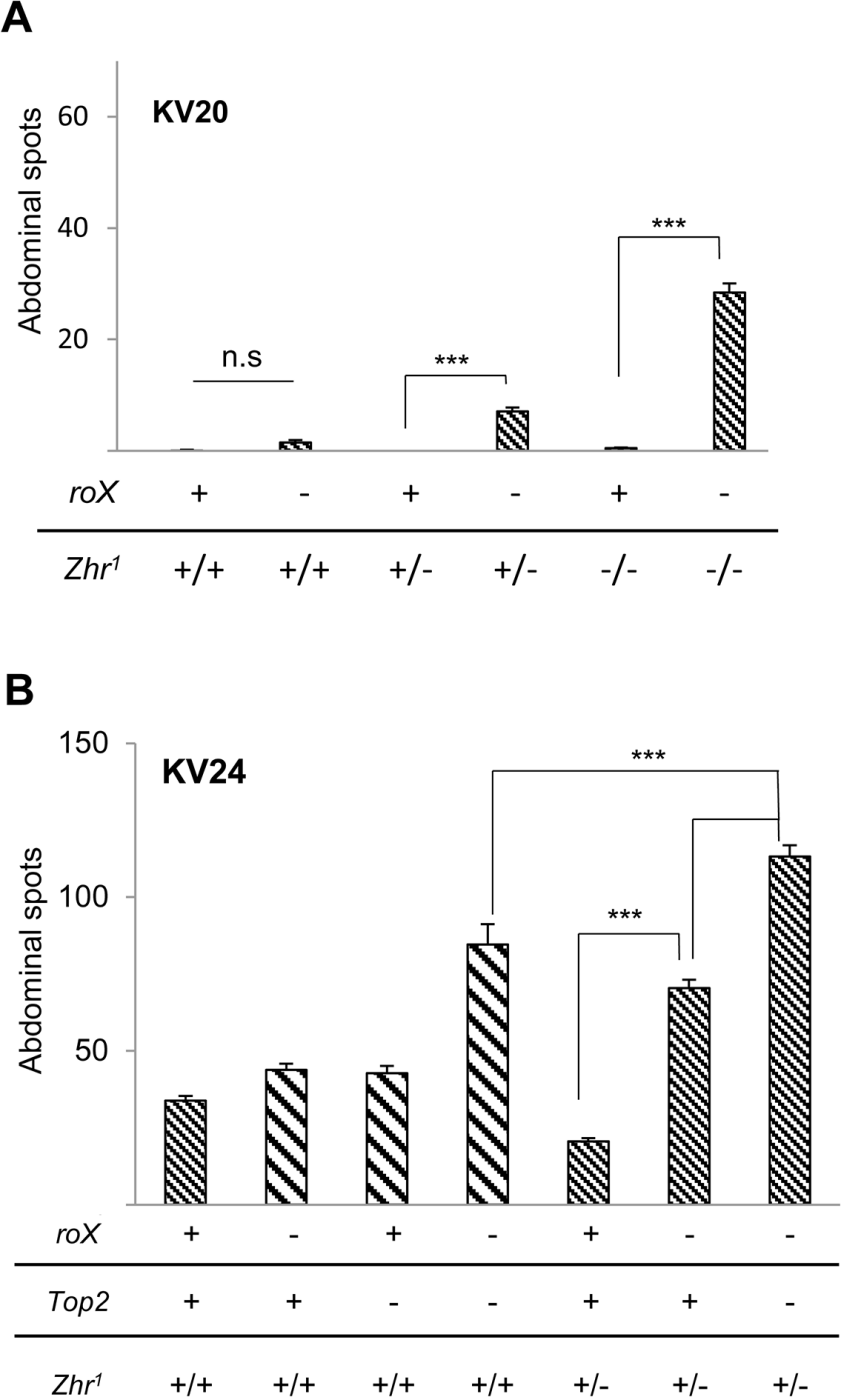
Pericentromeric X heterochromatin contributes to feminization of autosomal heterochromatin in XX flies. X heterochromatin was deleted by the X;Y translocation *Zhr* ^*1*^. (A) Females with one or two *Zhr* ^*1*^ chromosomes suppress PEV upon loss of *roX*. The KV20 reporter, which normally produces <1 spot/abdomen, was used. *roX* and *Top2* mutations are indicated by (-). Full genotypes (left to right): *yw*; KV20/+, *yw roX1* ^*ex33*^ *roX2Δ*; KV20/+, *yw/yw Zhr* ^*1*^; KV20/+, *yw roX1* ^*ex33*^ *roX2Δ* + / *yw roX1* ^*ex33*^ *roX2Δ Zhr* ^*1*^; KV20/+, *yw Zhr* ^*1*^ */yw Zhr* ^*1*^; KV20/+, *yw roX1* ^*ex33*^ *roX2Δ Zhr* ^*1*^ */ yw roX1* ^*ex33*^ *roX2Δ Zhr* ^*1;*^ KV20/+. Averages are derived from 20–50 flies of each genotype. *** indicates p-value <0.00001. (B) Loss of Top2 further masculinizes heterochromatin in *Zhr* ^*1*^/+ females. Greater suppression of PEV is observed in *roX* females mutated for *Top2* and with *Zhr* ^*1*^. This study uses the KV24 reporter, producing about 30 spots/female in a wild type background. Full genotypes (left to right): *yw*; KV24 /+, *yw roX1* ^*ex33*^ *roX2Δ*; KV24 /+, *yw*; *Top2* ^*17-1*^/*Top2* ^*17-3*^; KV24 /+, *yw roX1* ^*ex33*^ *roX2Δ*; *Top2* ^*17-1*^/ *Top2* ^*17-3*^; KV24 /+, *yw/yw Zhr* ^*1*^; KV24 /+, *yw roX1* ^*ex33*^ *roX2Δ Zhr* ^*1*^/ *yw roX1* ^*ex33*^ *roX2Δ* KV24 /+, *yw roX1* ^*ex33*^ *roX2Δ Zhr* ^*1*^/ *yw roX1* ^*ex33*^ *roX2Δ Top2* ^*17-1*^/ *Top2* ^*17-3*^; KV24 /+. Bars with coarse hatching are reproduced from Fig 4 for comparison. *** p-value <0.00001.

The involvement of *Top2* in a process that may be triggered by sex chromosome karyotype suggested an alternative mechanism. Over 10 Mb of X heterochromatin is composed of satellite repeats (359 bp repeats) that are unique to the X chromosome [39, 46]. Interestingly, the 359 bp repeats bind Top2 in interphase nuclei [11, 47]. This suggested the possibility that an interaction between X heterochromatin and Top2 determines differential heterochromatin sensitivity to loss of *roX*. If this is the case, deletion of X heterochromatin may act similarly to Top2 mutation. The X;Y translocation *Zhr* ^*1*^ replaces X heterochromatin with part of the Y chromosome [48, 49]. We generated *roX* mutant females that were heterozygous for *Zhr* ^*1*^ and carry KV20 (*yw roX1* ^*ex33*^ *roX2Δ Zhr* ^*1*^ */ yw roX1* ^*ex33*^ *roX2Δ +*; KV20/+). Interestingly, weak suppression of PEV was observed in *roX* females with a single *Zhr* ^*1*^ chromosome, but not in *Zhr* ^*1*^ females wild type for *roX* (Fig 5A). As removal of one copy of X heterochromatin generates XX females that now depend on *roX* for normal autosomal PEV, loss of X heterochromatin partially masculinizes autosomal heterochromatin in these flies.

The involvement of Top2 in homolog pairing, and its localization at the 359 bp repeats, suggested the possibility that a large block of unpaired 359 bp repeats itself could signal the XY karyotype. If this is the case, *Zhr* ^*1*^ */Zhr* ^*1*^ females, which have no unpaired 359 bp repeats, should display feminine heterochromatin. In contrast to this expectation, we found increased suppression of PEV in homozygous *Zhr* ^*1*^ females that lack *roX* (Fig 5A, right). However, no suppression of PEV was observed in homozygous *Zhr* ^*1*^ females with wild type *roX*. Suppression of PEV is thus not due solely to the differing chromatin content of *Zhr* ^*1*^ chromosomes. Our findings are consistent with an interaction between Top2 and X heterochromatin determining heterochromatin sensitivity to *roX*, but do not support the hypothesis that unpaired chromatin in the XY or XO nucleus is a factor.

The suppression of PEV in *roX* females with one or two *Zhr* ^*1*^ alleles is weak (contrast with suppression of PEV in *roX1 roX2* males, Fig 3B). To determine if the effects of *Top2* and *Zhr* ^*1*^ mutations are additive, we generated *Zhr* ^*1*^ */+* females mutated for Top2 and compared PEV in the presence and absence of *roX*. These females displayed greater suppression of PEV upon loss of *roX* than females mutated for *Zhr* ^*1*^ or *Top2* alone, supporting the idea that *Top2* and pericentric X heterochromatin act together (Fig 5B).

If the dose of X-heterochromatin acts as a signal for karyotype, duplication of this region in XY flies should feminize their heterochromatin. We attempted to generate XY flies with a duplication of X heterochromatin on the Y chromosome (*Zhr* ^*+*^ Y) to test this idea [11]. Unfortunately, no *roX1 roX2/ Zhr* ^*+*^ Y males were recovered, suggesting a genetic incompatibility between chromosomes in this mating.

## Discussion

Autosomal heterochromatin is typically not thought of as differing in males and females, but sexually dimorphic PEV has also been observed in mice, where a variegating transgene is more highly expressed in females [50]. This study found that both *SRY* and sex chromosome karyotype determine silencing. Importantly, this reveals that sexual dimorphism of autosomal heterochromatin is not limited to *Drosophila*. One attractive possibility is that both male and female flies require *roX* RNA for heterochromatic silencing, but male heterochromatin is more sensitive to loss of *roX*. The idea that *roX* RNAs might in fact also function in females is supported by the modest suppression of PEV sometimes observed in *roX1 roX2* females (Figs 4B, 5A and 5B). Although the *roX* RNAs are typically thought of as male-limited, *roX1* is abundantly expressed in early embryos of both sexes, and thus is available in females [51]. While we do not yet understand the rationale for the sex differences in autosomal heterochromatin in flies, the presence of a large, heterochromatic Y chromosome ensures that males have considerably more total heterochromatin than females. It is plausible that the chromatin content of XY cells drove a compensatory adaptation in male flies [8, 9].

The identification of *Top2* as a regulator of heterochromatic sexual dimorphism suggests that maintenance of normal chromatin organization plays a role in sex differences based on karyotype. However, the involvement of Top2 in numerous processes complicates analysis. For example, Top2 is itself required to maintain PEV in otherwise wild type males, but not in females. This provides additional evidence for the sexual dimorphism of autosomal heterochromatin, and is in agreement with a role for Top2 in chromatin condensation [52, 53]. However, it also suggests dual roles for Top2 in karyotype detection and heterochromatin maintenance.

Top2 has been reported to participate in the male-limited process of dosage compensation in studies using chemical inhibition or RNAi knockdown [45]. These manipulations produced a 2-fold reduction of expression in a plasmid-based model for dosage compensation. A physical association between Top2 and a single member of the MSL complex, the RNA/DNA helicase MLE, was also detected in these studies. Top2 has also been found with chromatin-bound MSL proteins in S2 cells, but, as Top2 is an abundant component of chromatin, this is unsurprising [54]. Our studies, performed with heteroallelic Top2 mutants, confirm a genetic interaction between MLE and Top2, but this appears equally important in males and females, and thus not limited to dosage compensation. The different methods by which Top2 activity was reduced in these studies may be responsible for this disparity. Interactions between helicases and Top2 are prevalent in other species. Yeast Top2 binds the Sgs1 helicase and mammalian Top2α interacts with BLM, the Bloom Syndrome helicase, and RNA helicase A, orthologous to MLE [55–57]. Disruption of the BLM-Top2α interaction leads to chromosome damage, and Top2 interaction with Sgs1 is required for decatenation *in vivo*. These interactions are thus important for genomic integrity. The nature of the Top2-MLE interaction remains an interesting question. *Drosophila* Top2 does associate with RNA, and it is possible that the helicase activity of MLE regulates this association [58]. We speculate that overexpression of MLE stabilizes mutant Top2 or supports its activity, increasing the survival of Top2 mutants of both sexes. An intriguing possibility, suggested by the association of the DEAD/H box RNA helicase P68 with mouse centromeric repeats, is that MLE promotes recruitment of Top2 to the 359 bp repeats [59].

The identification of Top2 as a pairing promoter suggested that X chromosome pairing could signal karyotype, but questions about the functions that are deficient in Top2 mutants complicate interpretation. Some function must be retained in *Top2* ^*17-1*^ */Top2* ^*17-3*^ mutants because adult escapers are recovered. However, embryos from *Top2* ^*17-1*^ */Top2* ^*17-3*^ mothers fail to initiate development, revealing a requirement for maternally deposited wild type Top2. It is possible that maternal Top2 is also sufficient to rescue near-normal pairing, transvection and insulation in *Top2* ^*17-1*^ */Top2* ^*17-3*^ flies. Indeed, studies with a similar heteroallelic Top2 combination found no defect in pairing of the 359 bp repeats [44]. This study, like ours, used larvae that received maternal Top2, potentially obscuring a requirement for Top2 in this process.

**Fig 6:**
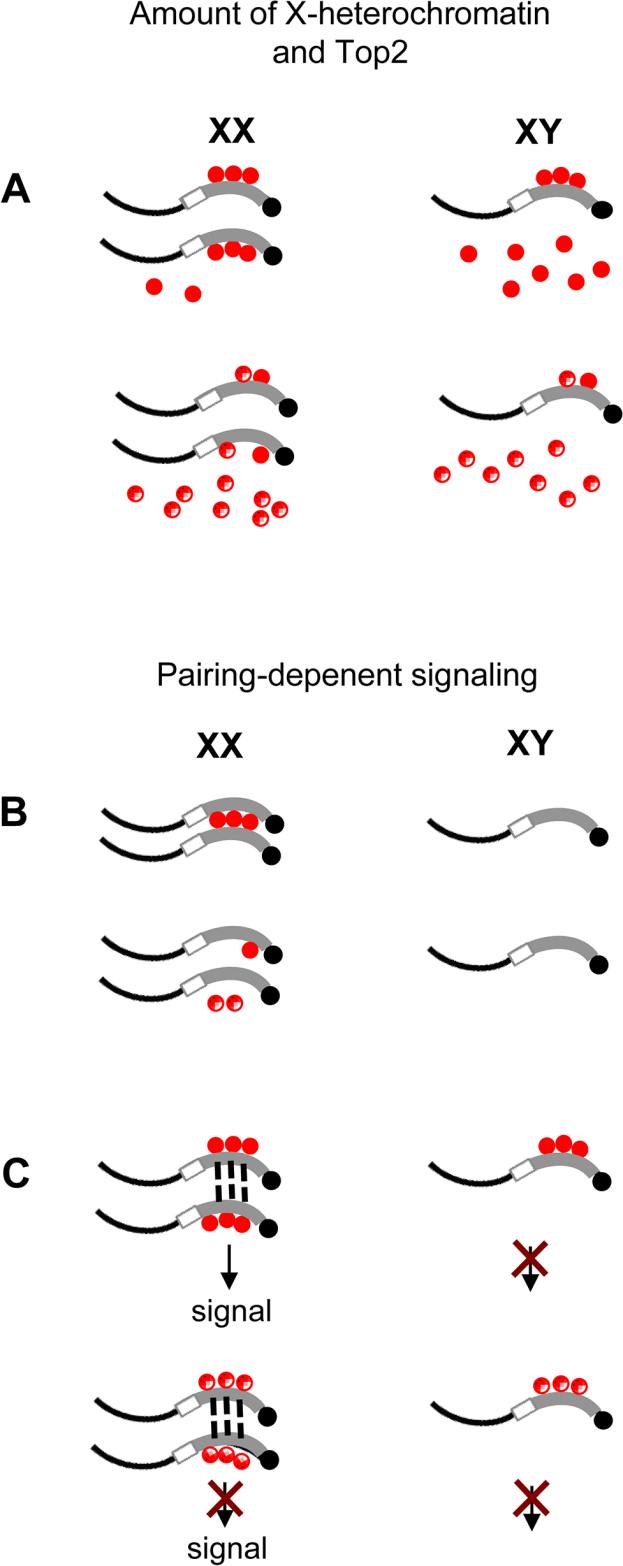
Models for detection karyotype detection. The absolute amount of X heterochromatin (A) or pairing of X heterochromatin (B, C) could generate a signal specifying the XX karyotype. XX flies have two copies of X heterochromatin (thick lines) but XY flies have one. Top2 (red) binds the 359 bp repeats (gray). (A) The absolute amount of Top2-bound 359 bp chromatin (top, left) or free Top2 in males (top, right) could generate a karyotype-specific signal. Mutant Top2 (A, bottom) is deficient in a function necessary for generation of the signal. Non-359 bp X-heterochromatin is shown in white. (B) Top2-dependent pairing of X heterochromatin could signal the XX karyotype (top). Mutant Top2 (bottom) fails to support normal pairing. (C) Top2-independent pairing of X-heterochromatin requires Top2 to generate or transmit a signal. *Top2* mutants (bottom) are deficient in this process.

*Top2* is enriched on the pericentric 359 bp repeats, and deletion of X-heterochromatin additively enhances masculinization of autosomal heterochromatin by *Top2* mutations. This prompted the idea that differences in karyotype may be detected by interaction of Top2 and a sequence within X-heterochromatin, possibly the 359 bp repeats. Several scenarios for how this might occur are possible. XX flies have double the X-heterochromatin of XY flies. An absolute difference in the amount of Top2-bound X heterochromatin could distinguish the male and female karyotypes (Fig 6A, left). It is also possible that higher free Top2 in males, with a single copy of the 359 bp repeats, is the source of a karyotype-specific signal (Fig 6A, right). This idea is supported by enhanced masculinization upon deletion of X heterochromatin. Although we obtained no evidence supporting the idea that unpaired chromatin signals the male karyotype, it remains possible that pairing of X heterochromatin, either dependent or independent of Top2, signals the XX karyotype (Fig 6B and 6C). For example, Top2-independent pairing of X-heterochromatin might occur, but association of Top2 with this region could be necessary to detect the paired status (Fig 6C).

Numerous sex determination strategies have arisen in heterogametic organisms. Each utilizes a primary signal that orchestrates the process of becoming female or male. Recent studies have highlighted the complexity of gene regulation at the bottom of the fly sex determination cascade [60–63]. In contrast, the chromosome counting mechanism at the top of the cascade was long thought to be the exclusive source of differences between the sexes [12, 31, 64]. Our findings suggest that the sex chromosomes of flies have additional ways of modulating phenotype. These findings are in accord with recent studies in multiple organisms documenting regulation by sex chromosome karyotype, rather than the conventional sex determination pathway (reviewed by [30]). Indeed, an analysis in fly heads revealed that most sex-biased gene regulation is not mediated by *tra* [61]. While some of this likely depends on upstream elements in the sex determination and dosage compensation cascade, the regulatory basis of a significant proportion of the genes identified by this study remains unknown. Our current findings are most easily interpreted as evidence that chromosome-specific repetitive sequences, and proteins that interact with these sequences, produce differences in the nuclear environment that reflect sex chromosome karyotype. We postulate that this leads to the differences in male and female autosomal heterochromatin that we have observed. The universality of repetitive sequences and Top2 in higher eukaryotes suggests a general mechanism that could operate in other heterogametic organisms.

## Materials and Methods

### Fly strains

Flies were maintained at 25°C on standard cornmeal–agar fly food. Unless otherwise noted, mutations are described in [65]. *roX1* mutations have been described [10, 29, 66]. Elimination of *roX2* was accomplished by a viable deletion (*roX2Δ*) or a lethal deletion complemented by a cosmid carrying essential genes but lacking *roX2* [10] [26]. Variegating insertions used as reporters in this study are described [67, 68]. A 4^th^ chromosome insertion of P[hsp26-pt, hsp70-*w*], marked with *w* ^*+mW*.*hs*^ (118E-10) and 2^nd^ (KV20) and 3^rd^ (KV24) chromosome insertions of P[SUPor-P], marked with *w* ^*+mC*^ and *yellow (y* ^*+*^) reporters were used. These reporters were selected to facilitate stock construction, but key findings were validated with multiple reporters. *Top2* ^*17-1*^ *and Top2* ^*17-3*^ mutations were generously provided by A. Hohl, C. T. Wu and P. Geyer [41]. Additional mutations are as follows: *Cap-D3* ^*c07081*^ [69], *Cap-H2* ^*Z0019*^ [70], *MCPH1* ^*0978*^ [71], *Dhc64c* ^*8-1*^ [72], [*w* ^*+*^-hsp83 MLE] [73], [*w* ^*+*^-hsp83 MSL2]6I and [*w* ^*+*^-hsp83 MSL1]Z1 [23, 74], 2X*P(w* ^+mC^,*sisA* ^+^)+2X*P(w* ^+mC^,*sc* ^sisB+^) [36, 75]. Descriptions of *Sxl* ^*2593*^, *Sxl* ^*M1F3*^, *Tra2* ^*B*^, *Tra2* ^*ts1*^, *Dsx* ^*1*^, *Dsx* ^*D*^, *Top2* ^*17-1*^, *Top2* ^*17-3*^, *Cap-D3* ^*c07081*^, *Cap-H2* ^*Z0019*^, *MCPH1* ^*0978*^, *Dhc64c* ^*6-10*^, *Dhc64c* ^*8-1*^, *fs(1)h* ^*1*^, *Zhr* ^*+*^ Y and *Zhr* ^*1*^ are available on Flybase (http://www.flybase.org). All other strains used in this study were obtained from the Bloomington Drosophila Stock Center.

### Transvection and insulator assays

Restoration of pigmentation by transvection at *y* is a standard measure of homolog pairing [76–78]. Pigmentation was scored in 1–2 days old flies on a scale of 1–4, where 1 is the no pigmentation and 4 is wild type levels. At least 100 flies of each genotype were scored. The *y* ^*2*^ *Gypsy* insertion contains an insulator that disrupts communication between the *y* enhancer and promoter [76]. Flies were aged for 24 h before scoring on the pigmentation scale described above. At least 25 flies from two independent crosses were scored. Significance was determined by a Student’s T-test. Images were obtained using a Zeiss Discovery V8 stereo microscope.

## Supporting Information

S1 Fig Suppression of PEV in *roX1 roX2* males is independent of reporter or insertion site. PEV of *y* ^*+*^ in KV24 (3^rd^ chromosome) is visible as black abdominal spots in both sexes and is suppressed in *roX* males (top), but not in *roX* females (bottom). PEV of *w* ^*+mW*.*hs*^ in 118E-10 (4^th^ chromosome) is detected by eye pigmentation. *roX* males (top), but not females (bottom), suppress 118E-10 PEV. 118E-10 was examined in the *yw roX1* ^*ex33*^ *Df(1)52*;[4*Δ*4.3]/+ background, which is mutated for *roX1* and *roX2* and lacks other *w* markers, enabling visualization of the *w* ^*+mW*.*hs*^ reporter.
(PDF)

S2 Fig Pairing regulators that do not affect heterochromatic sex. Heterochromatic sex was determined in flies mutated for anti-pairers (*Cap-H2*, *Cap-D3* and *fs(1)h*) and pairing promoters (*MCPH1* and *Dhc64c*). All flies carried the *y* ^*+*^ KV20 reporter. Flies mutated for each pairing regulator were generated in wild type (++) and *yw roX1* ^*ex33*^ *roX2Δ* mutant backgrounds. Almost no abdominal pigmentation was observed in XX flies wild type (white) or mutated (hatched) for both *roX* genes. In contrast, PEV in XY flies (black) is suppressed in *roX* mutants (dark gray). A slight enhancement of PEV is detected in *Cap-D3* mutant flies, consistent with previous reports of condensin mutations as PEV enhancers [79, 80]. Fifteen-50 flies were counted for each genotype.
(PDF)

S3 Fig *Top2* ^*17-1*^ */Top2* ^*17-3*^ mutants are deficient in specific functions. A) The *Top2* mutations disrupt different domains. Missense mutations *Top2* ^*17-1*^ (WHD domain) and *Top2* ^*17-3*^ (TOPRIM domain). B) *Top2* ^*17-1*^ */Top2* ^*17-3*^ males are fertile but *Top2* ^*17-1*^ */Top2* ^*17-3*^ females are sterile. Both mutations are homozygous lethal. C) Characteristic abnormalities in a polytene preparation from a *Top2* ^*17-1*^ */Top2* ^*17-3*^ male larvae. A puffy X chromosome (black arrow) and homolog unpairing (white arrows) are visible. One hundred-250 nuclei from at least 5 larvae were scored for each genotype. D) Transvection restores *yellow* expression. *y* ^*82f29*^ is a deletion of upstream enhancer elements. *y* ^*1#8*^ retains enhancers but lacks a promoter. *y* ^*3c3*^ lacks a bristle enhancer and the promoter, but retains a wing enhancer. Pairing between *y* ^*82f29*^ and *y* ^*1#8*^ or *y* ^*3c3*^ enables enhancers on the homolog to drive the *y* ^*82f29*^ promoter, restoring expression. Drawing based on [77]. Wing and body pigmentation was ranked from 1 (no pigmentation) to 4 (wild type). Flies homozygous for each allele have light body and wing color (1,1). Transvection in *y* ^*82f29*^ */y* ^*1#8*^ flies restores wing and body color near wild-type levels (3, 3). Transvection in *y* ^*82f29*^ */y* ^*3c3*^ flies restores wing pigmentation only (3, 1). Transvection is not disrupted in *Top2* ^*17-1*^ */Top2* ^*17-3*^ mutants (shaded). Flies were aged 1–2 days before scoring and photography. At least 100 flies were scored for each genotype. E) Representative abdomens showing *y* transvection. Full genotypes are: *y* ^*82f29*^ */y* ^*1#8*^; *Top2* ^*m*^ */ Cyo*, *y* ^*82f29*^ */y* ^*1#8*^; *Top2* ^*17-1*^ */Top2* ^*17-3*^. F) *Top2* mutations do not disrupt *Gypsy* insulation. Loss of pigmentation in *y* ^*2*^ requires the Top2-dependent *Gypsy* insulator. Loss of insulation enhances body pigmentation. Full genotypes are: *y* ^*2*^/Y; *+/+*, *y* ^*2*^/Y; *Top2* ^*m*^ */CyO* and *y* ^*2*^/Y; *Top2* ^*17-1*^ */Top2* ^*17-3*^. At least 25 flies of each genotype were aged for 24 h before scoring.
(PDF)

S1 Table Polytene preparations from Top2 mutants display altered X-chromosome morphology and disrupted pairing. Polytene preparations from control (+/+, reference *yw* strain) and *yw; Top2* ^*17-1*^/ *Top2* ^*17-3*^ larvae were examined for disrupted morphology and local unpairing. The incidence of abnormality, and total nuclei scored, is in parentheses. Chromosomes with a diffuse banding pattern and those bloated along the entire chromosome length were scored as abnormal. Nuclei with any visible unpairing of homologs was scored as positive for unpairing.
(PDF)
