# Supplementary figures and images for: Sex Differences in Drosophila melanogaster Heterochromatin Are Regulated by Non-Sex Specific Factors
Source: PLoS One. 2015 Jun 8;10(6):e0128114. doi: 10.1371/journal.pone.0128114 (PMC4459879; doi:10.1371/journal.pone.0128114)

Suppression of PEV in *roX1 roX2* males is independent  
of insertion site or reporter

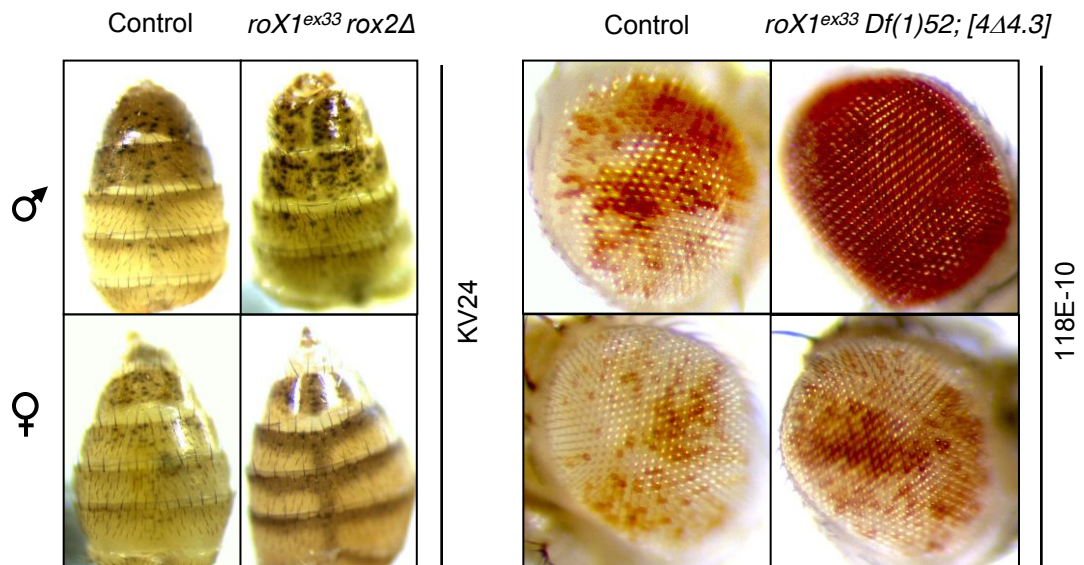

Supplement: S1 Fig — PEV of y + in KV24 (3rd chromosome) is visible as black abdominal spots in both sexes and is suppressed in roX males (top), but not in roX females (bottom). PEV of w +mW.hs in 118E-10 (4th chromosome) is detected by eye pigmentation. roX males (top), but not females (bottom), suppress 118E-10 PEV. 118E-10 was examined in the yw roX1 ex33 Df(1)52;[4Δ4.3]/+ background, which is mutated for roX1 and roX2 and lacks other w markers, enabling visualization of the w +mW.hs reporter. (PDF) [file pone.0128114.s001.pdf]

Pairing regulators that do not influence heterochromatic sex

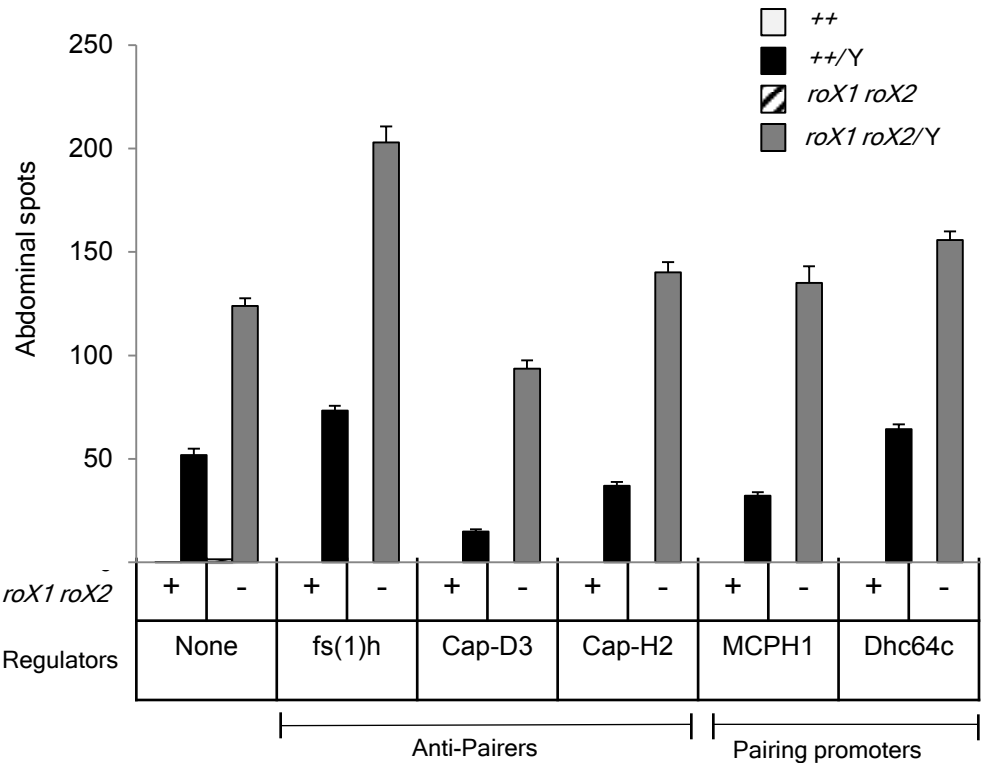

Supplement: S2 Fig — Heterochromatic sex was determined in flies mutated for anti-pairers (Cap-H2, Cap-D3 and fs(1)h) and pairing promoters (MCPH1 and Dhc64c). All flies carried the y + KV20 reporter. Flies mutated for each pairing regulator were generated in wild type (++) and yw roX1 ex33 roX2Δ mutant backgrounds. Almost no abdominal pigmentation was observed in XX flies wild type (white) or mutated (hatched) for both roX genes. In contrast, PEV in XY flies (black) is suppressed in roX mutants (dark gray). A slight enhancement of PEV is detected in Cap-D3 mutant flies, consistent with previous reports of condensin mutations as PEV enhancers [79, 80]. Fifteen-50 flies were counted for each genotype. (PDF) [file pone.0128114.s002.pdf]

Characterization of Top2 mutant

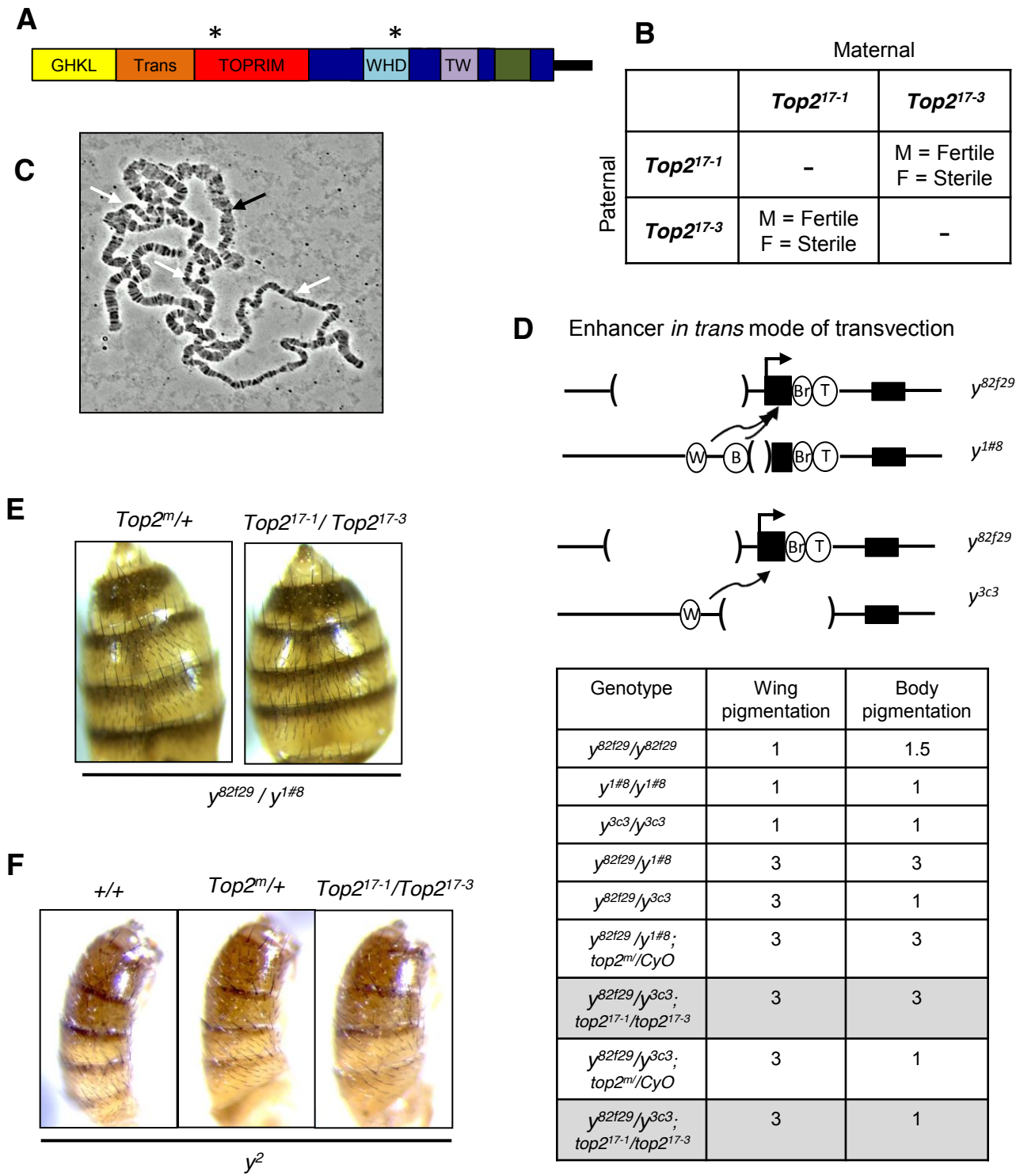

Supplement: S3 Fig — A) The Top2 mutations disrupt different domains. Missense mutations Top2 17-1 (WHD domain) and Top2 17-3 (TOPRIM domain). B) Top2 17-1 /Top2 17-3 males are fertile but Top2 17-1 /Top2 17-3 females are sterile. Both mutations are homozygous lethal. C) Characteristic abnormalities in a polytene preparation from a Top2 17-1 /Top2 17-3 male larvae. A puffy X chromosome (black arrow) and homolog unpairing (white arrows) are visible. One hundred-250 nuclei from at least 5 larvae were scored for each genotype. D) Transvection restores yellow expression. y 82f29 is a deletion of upstream enhancer elements. y 1#8 retains enhancers but lacks a promoter. y 3c3 lacks a bristle enhancer and the promoter, but retains a wing enhancer. Pairing between y 82f29 and y 1#8 or y 3c3 enables enhancers on the homolog to drive the y 82f29 promoter, restoring expression. Drawing based on [77]. Wing and body pigmentation was ranked from 1 (no pigmentation) to 4 (wild type). Flies homozygous for each allele have light body and wing color (1,1). Transvection in y 82f29 /y 1#8 flies restores wing and body color near wild-type levels (3, 3). Transvection in y 82f29 /y 3c3 flies restores wing pigmentation only (3, 1). Transvection is not disrupted in Top2 17-1 /Top2 17-3 mutants (shaded). Flies were aged 1–2 days before scoring and photography. At least 100 flies were scored for each genotype. E) Representative abdomens showing y transvection. Full genotypes are: y 82f29 /y 1#8; Top2 m / Cyo, y 82f29 /y 1#8; Top2 17-1 /Top2 17-3. F) Top2 mutations do not disrupt Gypsy insulation. Loss of pigmentation in y 2 requires the Top2-dependent Gypsy insulator. Loss of insulation enhances body pigmentation. Full genotypes are: y 2/Y; +/+, y 2/Y; Top2 m /CyO and y 2/Y; Top2 17-1 /Top2 17-3. At least 25 flies of each genotype were aged for 24 h before scoring. (PDF) [file pone.0128114.s003.pdf]
