# Supplementary material for: Sex Differences in Drosophila melanogaster Heterochromatin Are Regulated by Non-Sex Specific Factors
Source: PLoS One. 2015 Jun 8;10(6):e0128114. doi: 10.1371/journal.pone.0128114 (PMC4459879; doi:10.1371/journal.pone.0128114)
Supplement: S1 Table — Polytene preparations from control (+/+, reference yw strain) and yw; Top2 17-1/ Top2 17-3 larvae were examined for disrupted morphology and local unpairing. The incidence of abnormality, and total nuclei scored, is in parentheses. Chromosomes with a diffuse banding pattern and those bloated along the entire chromosome length were scored as abnormal. Nuclei with any visible unpairing of homologs was scored as positive for unpairing. (PDF) [file pone.0128114.s004.pdf]

Polytene chromosome structure in *Top2* mutants

Genotype	Karyotype	Normal X	Abnormal or puffy X	Unpairing
+/+	XX	**95.26%** (161/169)	**4.74%** (8/169)	**13.01%** (22/169)
	XY	**85.53%** (136/159)	**14.47%** (24/159)	**15.72%** (25/159)
$Top2^{17-1}$/$Top2^{17-3}$	XX	**87.02%** (208/239)	**12.97%** (31/239)	**47.69%** (114/239)
	XY	**29.63%** (72/243)	**70.37%** (171/243)	**54.73%** (133/243)
